# Drug Tolerance in Biomembranes

**Published:** 1995

**Authors:** Emanuel Rubin

**Affiliations:** Emanuel Rubin, M.D., is the Gonzalo E. Aponte Professor of Pathology and chairman of the Department of Anatomy, Pathology, and Cell Biology, Jefferson Medical College, Philadelphia, Pennsylvania

**Keywords:** AOD tolerance, cell membrane, AOD effects (AODE), membrane fluidity

**Figure:**
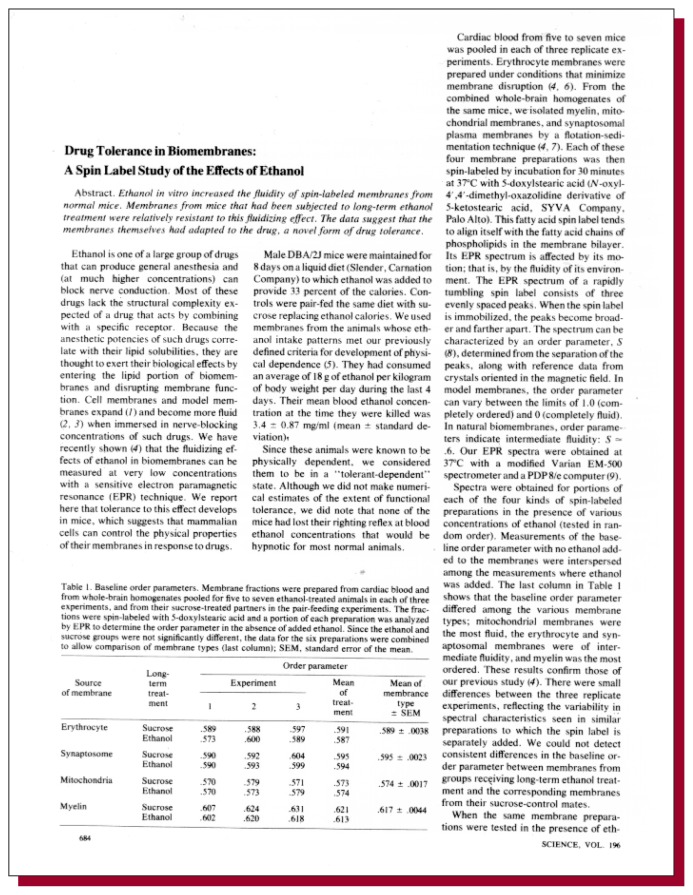
Chin, J.H., and Goldstein, D.B. Drug tolerance in biomembranes: A spin label study of the effects of ethanol. *Science* 196(4290):684–685, 1977.

Behavioral tolerance to drugs, particularly alcohol, has been part of folklore for most of recorded history; it is common wisdom that some who drink heartily and frequently are better able to “hold their liquor” than are others who consume a thimbleful of sacramental wine during the year. The mechanism(s) of alcoholic intoxication, let alone tolerance, still are not understood, although many researchers have thought that alcohol affects the central nervous system in a manner similar to that of inhalation anesthetics. Anesthetic effects are governed by the Meyer-Overton rule, which in its original formulation almost a century ago held that the potency of a general anesthetic is directly correlated with its lipid solubility (i.e., how readily it dissolves in tissues containing fats). Thus, the more soluble an anesthetic is in lipids, the more potent are its effects. With the development of cell biology in the 1960’s, it became clear that the lipids of the Meyer-Overton law are the phospholipids (i.e., a type of lipid, or fat) that forms the structural basis of cell membranes. Further progress in this area came in the late 1960’s, when Hubbell and McConnell (1969) demonstrated that general anesthetics, including alcohol, cause molecular disordering of biological membranes. This article and subsequently those of other researchers (e.g., Trudell et al. 1973) suggested that alcohol exerts its intoxicating effects by dissolving in the membrane and “fluidizing” that structure, thereby in some way altering the function of the cell.

Although the aforementioned studies were relevant to the study of anesthesia and alcohol intoxication, they did not address the problem of behavioral tolerance to alcohol and thus seemed distant from the concerns of alcoholism or chronic alcohol abuse. It was in this context that Chin and Goldstein (1977) were able to link, for the first time, the induction of a “tolerant-dependent” state to changes in a physical parameter. These authors demonstrated that portions of the membranes of nerve terminals (i.e., synaptosomes) from mice treated chronically with alcohol until they had reached a state of alcohol tolerance and physical dependence were resistant to the fluidizing effect of alcohol in vitro. In Chin and Goldstein’s study, the molecular order, or “fluidity,” of the isolated membranes in vitro was unchanged by chronic alcohol intake as long as alcohol was not present in the solution. However, when alcohol was added to the medium, the expected fluidizing response was blunted.

This seminal article actually had wider implications than the link established between a behavioral phenomenon and a biological alteration. It also demonstrated in general that mammalian cells can adapt to perturbing conditions by modulating the “fluidity” (and by inference the composition) of their membranes.

The study by Chin and Goldstein stimulated numerous other studies of “membrane tolerance.” The resistance to fluidization was later extended to include other membranes as well as components of liver and pancreatic cells (Taraschi and Rubin 1985). The resistance also was found to be associated with a decrease in the solubility of alcohol and other anesthetics in membranes (Rottenberg et al. 1981). It was further demonstrated that artificial membranes composed of phospholipids purified from cells of chronically intoxicated rats also are resistant to disordering by alcohol and that the property of “membrane tolerance” seems to reside particularly in anionic (negatively charged) phospholipids (such as phosphatidylinositol, phosphatidylserine, and cardiolipin) (Taraschi et al. 1986).

As is the case in many fields, the original concept that anesthetics (and alcohol) produce their effects on the central nervous system solely by interacting with membrane lipids now appears too simplistic. Increasing evidence exists that alcohol interacts directly with proteins embedded in or associated with cell membranes (Franks and Lieb 1994; Slater et al. 1993; Li et al. 1994). These interactions also follow the Meyer-Overton rule, which can be reinterpreted to substitute interactions between alcohol and these proteins for lipid solubility. Yet direct alcohol-protein interactions do not (at least currently) explain behavioral tolerance, and modulations of such interactions by membrane lipids have been demonstrated (Slater et al. 1993). Thus, lipid-protein interactions remain to be explored, and the adaptive lipid response demonstrated by Chin and Goldstein may yet prove to regulate the development of behavioral tolerance to alcohol.
